# Decent Work Mediates the Relationship Between Work Capital and Work Engagement Among Nurses

**DOI:** 10.1002/nop2.70633

**Published:** 2026-06-08

**Authors:** Liping Lin, QiQi Zhang, Yvxvan Li, Tao Zhou, Lili Yang, Jiekai Yang, Shuyan Zhang, Lihui Yan, Qi Zhou

**Affiliations:** ^1^ Hangzhou Linping District Integrated Traditional Chinese and Western Medicine Hospital Hangzhou China; ^2^ Shaoxing Central Hospital ShaoXing China; ^3^ School of Statisitcs and Data Science, Shanghai University of Finance and Economics Shanghai China; ^4^ Department of Emergency Affliated Hangzhou First People's Hospital, School of Medicine, Westlake University Hangzhou China; ^5^ Sir Run Run Shaw Hospital of Zhejiang University, School of Medicine Hangzhou China; ^6^ The Second Clinical College, Zhejiang Chinese Medical University Hangzhou China; ^7^ Nursing Department Hangzhou Linping District Integrated Traditional Chinese and Western Medicine Hospital Hangzhou China

**Keywords:** decent work, job demands–resources model, job resources, mediation analysis, nursing, work capital, work engagement

## Abstract

**Background:**

Work engagement is essential for nurses' motivation, retention and quality of care. However, the mechanisms linking organizational resources to engagement remain insufficiently understood. Grounded in the Job Demands–Resources (JD‐R) model, this study examined whether decent work is statistically associated with the relationship between work capital (WC) and work engagement among nurses.

**Methods:**

A cross‐sectional survey was conducted among 535 nurses from multiple hospitals in Zhejiang Province, China. Standardized instruments were used to assess WC, Decent Work (DW), and Work Engagement (UWES). Data were analysed using confirmatory factor analysis and mediation analysis (PROCESS Model 4, 5000 bootstrap samples).

**Results:**

Work Capital was positively associated with decent work (B = 0.74, *p* < 0.001) and work engagement (B = 0.19, *p* < 0.001). Decent work was positively associated with work engagement (B = 0.85, *p* < 0.001). Mediation analysis indicated a significant indirect association (indirect effect = 0.67, 95% CI [0.56, 0.79]), accounting for 76.1% of the total association. Subscale comparisons, based on standardized mean scores, suggested relatively higher dedication and lower absorption levels.

**Conclusion:**

Decent work may function as a potential explanatory pathway linking work capital and work engagement. These findings highlight the importance of considering both resource availability and perceived work quality when examining engagement in nursing contexts.

**Impact:**

This study extends the JD‐R framework by examining decent work as a potential mediating mechanism between work capital and work engagement in nurses.

**Reporting Method:**

This study followed the STROBE guidelines for cross‐sectional studies.

**Patient or Public Contribution:**

No patient or public contribution.

## 1 Background

Work engagement (WE) has emerged as a central topic in contemporary nursing and organizational psychology research. It is widely defined as a positive, fulfilling and enduring psychological state that employees exhibit toward their work, characterized by three dimensions: vigour, dedication and absorption (Schaufeli et al. 2002; Antoinette 2012). Extensive evidence has demonstrated that work engagement is closely associated with job satisfaction, organizational commitment, turnover intention and overall organizational performance (Wei et al. 2023). In the nursing field, nurses' work engagement not only shapes their professional experience but also directly affects care quality, patient safety and the continuity of healthcare services (Muroi et al. 2023). However, empirical findings indicate that nurses generally report moderate to moderately high levels of engagement, with significant variations across departments, regions, and career stages (Aydogdu 2024). Notably, during and after the COVID‐19 pandemic, heightened job stress and burnout have substantially undermined nurses' sustained engagement, mainly due to excessive workloads and limited resources (Rizzo et al. 2023). Thus, under the global shortage of nursing personnel, examining the determinants and mechanisms of nurses' work engagement holds profound theoretical and practical significance.

Work Capital provides a valuable lens for understanding nurses' work engagement. It encompasses the array of capital resources that employees can access and mobilize in their work, including human capital (professional skills and knowledge), social capital (collegial relationships and team support), institutional capital (organizational policies and career development opportunities) and material capital (equipment and information systems) (Kim and Allan 2024). In nursing research, related concepts such as “nursing intellectual capital” and “workplace social capital” have been widely examined. Empirical studies have shown that these capital resources are significantly linked to job satisfaction, turnover intention, care quality and organizational performance among nurses (Xu, Cao, et al. 2024; Pedersen et al. 2023). Nevertheless, disparities in the distribution and utilization of Work capital persist across hospitals and departments, with issues such as understaffing, limited training opportunities and poor communication undermining nurses' clinical effectiveness and engagement levels. Recently, Yakusheva proposed the ‘nursing human capital value model’, which underscores institutional investments—such as continuing education and career pathways—as key drivers for enhancing organizational work capital (Yakusheva et al. 2024). However, further empirical validation and cross‐cultural adaptation of this model remain necessary.

Despite extensive research demonstrating that job resources are positively associated with work engagement, the mechanisms through which different types of resources translate into motivational outcomes remain insufficiently specified, particularly in nursing contexts characterized by high demands and constrained resources (Feng et al. 2025; BowenXue et al. 2024). In this study, work capital is conceptualized as the objective availability of economic, human, social and cultural resources (Wei et al. 2023; Pedersen et al. 2023), whereas decent work reflects nurses' subjective evaluation of the quality, fairness and dignity of their work conditions (Duffy et al. 2017; Blustein et al. 2023). Although both constructs are related to job resources, they represent distinct levels of analysis—resource availability versus perceived work quality. Within the Job Demands–Resources (JD‐R) framework, job resources are typically modelled as direct predictors of engagement; however, recent perspectives suggest that employees' interpretation and internalization of resources may constitute a critical intermediate process (Bakker et al. 2014). At the same time, it is important to acknowledge that alternative explanations are plausible (Tang et al. 2022). Job demands, organizational support, leadership and burnout may independently or jointly influence both decent work perceptions and engagement (Duffy et al. 2017).

Therefore, this study aims to examine a theoretically grounded but empirically underexplored pathway in which work capital is associated with work engagement through decent work as a perceptual mechanism while recognizing that this represents one of several plausible models rather than a definitive causal structure.

## 2 Relationships Among Variables and Theoretical Framework

### 2.1 Work Capital and Nurses' Work Engagement

According to the JD‐R model, abundant job resources enable employees to sustain and enhance their work engagement. For nurses, higher levels of Work Capital—such as adequate equipment, supportive work environments and opportunities for professional growth—can alleviate workload and foster greater enthusiasm toward their work. Work capital encompasses the resources employees possess in their professional settings, including material assets, knowledge, skills and emotional support. These resources effectively reduce occupational stress and strengthen work motivation. Recent studies have shown that sufficient Work Capital significantly enhances nurses' engagement (Saraswati and Pertiwi 2020; Bonner 2016; Tan et al. 2020). Specifically, when nurses have access to career development opportunities, continuous education and emotional support, their psychological capital is strengthened, empowering them to manage work‐related challenges and maintain a high level of engagement (Caesens et al. 2014).

Hypothesis 1 *Work Capital is positively associated with nurses' work engagement*.

### 2.2 Decent Work and Nurses' Work Engagement

Within the JD‐R framework, Decent Work provides employees with essential resources—such as job security, social support and developmental opportunities—that not only buffer work‐related stress but also boost motivation and positive affect, thereby enhancing engagement. Specifically, Decent Work helps nurses mitigate burnout and fosters greater enthusiasm and commitment to their roles. As a multidimensional construct, Decent Work encompasses fair treatment, a safe work environment and career advancement opportunities. Empirical studies indicated that Decent Work significantly improves job satisfaction and engagement. In the nursing context, a supportive work environment, fair compensation and a sense of professional security directly influence nurses' engagement levels (Strömgren et al. 2016).

Hypothesis 2 *Decent Work is positively associated with nurses' work engagement*.

### 2.3 Relationship Between Work Capital and Decent Work

The JD‐R model posits that organizational resource allocation determines employees' access to job resources. By providing sufficient work capital, organizations can create favourable working conditions, reduce job stress and offer career development opportunities, thereby cultivating an environment conducive to decent work (Xue et al. 2025). Adequate work capital serves as the foundation for achieving decent work. Research has demonstrated that organizational resource configurations—including material resources, training opportunities and team support—directly influence the realization of decent work (Sönmez et al. 2023; Zheng et al. 2024). Work capital encompasses not only tangible assets but also emotional and social resources, all of which are essential components of decent work (Ferraro et al. 2018; Zheng et al. 2025).

Hypothesis 3 *Work capital is positively associated with decent work*.

### 2.4 The Mediating Role of Decent Work Between Work Capital and Nurses' Work Engagement

While both work capital and decent work can be conceptualized as forms of job resources, they differ in their functional roles within the JD‐R framework (Bakker et al. 2014). Work Capital represents the availability of resources provided by the organization and accessible to employees, whereas decent work reflects employees' subjective appraisal of whether these resources translate into fair, secure and meaningful working conditions. From a theoretical perspective, it is plausible that employees first access or perceive available resources (work capital), and subsequently evaluate the extent to which these resources meet their expectations for dignity, fairness and well‐being (decent work), which then influences motivational outcomes such as engagement. This interpretation aligns with emerging extensions of the JD‐R model that emphasize cognitive and affective appraisal processes as intermediaries between resources and motivation (Zheng et al. 2025).

However, alternative model specifications are also possible. Decent Work could function as a moderator, strengthening or weakening the association between work capital and engagement. Similarly, reverse or reciprocal relationships may exist, whereby highly engaged nurses perceive their work conditions more positively. Given these possibilities, the present study adopts a mediational model as a theoretically informed but not exclusive representation of the relationships among variables, which should be interpreted cautiously.

Hypothesis 4 *Decent Work is statistically associated with the relationship between Work Capital and nurses' Work Engagement*.

## 3 Methods

### 3.1 Participants

This study employed a cross‐sectional survey design. Data were collected between 8/2025–11/2025 from nurses working in six hospitals across Zhejiang Province, China, including three tertiary hospitals, two secondary hospitals, and one primary hospital, representing a range of healthcare settings. A convenience sampling strategy was used. Within each participating hospital, nurses were recruited from both general units (e.g., internal medicine and surgery) and specialized units (e.g., intensive care, emergency and perioperative units) to enhance variability in work environments. Inclusion criteria were: (1) registered nurses with ≥ 1 year of clinical experience; (2) at least 6 months of continuous employment in the current department; and (3) voluntary participation. Exclusion criteria included: (1) nursing interns or trainees; (2) nurses on extended leave during the study period; and (3) administrative staff without direct patient care responsibilities.

Data were collected an anonymous online questionnaire distributed via hospital coordinators. To minimize duplicate responses, each participant could submit the survey only once device‐based restrictions. Incomplete questionnaires (missing > 10% of items) were excluded from analysis (Constantin et al. 2023). A total of 593 questionnaires were distributed, and 535 valid responses were retained (response rate = 90.2%). While convenience sampling limits generalizability, efforts were made to include diverse hospital types and departments to improve sample heterogeneity (Hayes and Scharkow 2013).

### 3.2 Measures

The survey included standardized scales to measure WC, DW and UWES. It also collected demographic information, including sex, education, work years, title and age.

#### 3.2.1 Work Capital

We used Kim et al.'s 16‐item scale to measure work capital (Kim and Allan 2024). The scale uses a Likert 7 scale with a total score of 16–112, with higher scores indicating a higher level of work capital. The questionnaire is divided into four dimensions: economic, human, social and cultural. One sample item is I can afford to enrol in job training programs. Total Cronbach's alpha = 0.90; Economy: 0.86, Manpower: 0.91, society: 0.85, Culture: 0.83 (Kim and Allan 2024).

#### 3.2.2 Decent Work

Decent Work was assessed using Duffy's 15‐item decent work scale (Duffy et al. 2017). The scale employs a 7‐point Likert format, yielding a total score ranging from 15 to 105, with higher scores indicating higher levels of Decent Work. It comprises five dimensions: Workplace Emotional Safety and Absence of Abuse, Healthcare Benefit Provision, Pay Fairness and Adequacy, Work–Life Balance and Leisure Time Assurance and Value Alignment. A sample item is: ‘My hospital provides me with an excellent healthcare plan’. The Cronbach's α coefficients were 0.90 for the total scale, and 0.79, 0.97, 0.87, 0.87, and 0.95 for the five subscales, respectively.

#### 3.2.3 Work Engagement

Work engagement was measured using the 15‐item Utrecht Work Engagement Scale (UWES) developed by Schaufeli et al. (Schaufeli et al. 2002). The Chinese version, translated and validated by Liu Chaoying, has been widely applied in nursing populations (Liu et al. 2013). The scale includes three dimensions: Vigour, Dedication and Absorption. Each item is rated on a 7‐point Likert scale, with total scores ranging from 0 to 90, where higher scores reflect greater work engagement. A sample item is: ‘At my work, I feel bursting with energy’. Luo Wen et al. tested the reliability and validity of this scale among nurses, reporting a Cronbach's α of 0.93 for the total scale and 0.87, 0.84, and 0.81 for the three subscales, respectively (Schaufeli et al. 2002).

All measurement scales underwent translation and back‐translation for linguistic accuracy. Panels of nursing experts checked each item for cultural relevance. Pilot testing with 30 older adults confirmed clarity and reliability. All adapted scales demonstrated internal consistency above 0.85, indicating strong reliability.

Regarding instrument adaptation, the Chinese version of the UWES has been previously validated in nursing populations and was used without modification. The work capital and decent work scales were translated into Chinese following a standard forward–backward translation procedure, involving bilingual experts and independent reviewers to ensure semantic equivalence. A pilot study was conducted with 30 clinical nurses to assess clarity, comprehension and contextual relevance. Minor wording adjustments were made accordingly. All adapted scales demonstrated internal consistency above 0.85, indicating strong reliability. Internal consistency reliability was assessed in the present sample. Cronbach's α coefficients for the retained items were 0.91 for Work Capital, 0.93 for Decent Work, and 0.92 for Work Engagement, indicating high reliability.

### 3.3 Statistical Analysis

All statistical analyses were conducted using SPSS 25.0 and R (lavaan package). First, data screening procedures were performed. Missing data were minimal (< 2%) and handled with mean imputation at the item level. Outliers were assessed using standardized z‐scores (|z| > 3.29), with no extreme cases identified. Normality was evaluated using skewness and kurtosis, which were within acceptable thresholds (|skewness| < 2, |kurtosis| < 7), supporting the use of parametric analyses. To assess common method bias (CMB), Harman's single‐factor test was conducted. The first unrotated factor accounted for 34.2% of the total variance, below the critical threshold of 40%, suggesting that CMB was not a serious concern. To assess the robustness of the findings, an additional sensitivity analysis was conducted using the original full‐scale composite scores prior to CFA‐based item refinement. The mediation pattern and statistical significance of indirect effects remained consistent with the primary analyses based on retained items.

Next, confirmatory factor analysis (CFA) was conducted using maximum likelihood estimation to evaluate the measurement model. Items with standardized factor loadings below 0.60 or with high residuals were considered for removal, guided by both statistical criteria and theoretical relevance. Model fit was evaluated using multiple indices: χ^2^/df < 3, CFI and TLI ≥ 0.90, RMSEA ≤ 0.08, and SRMR ≤ 0.08. Following CFA refinement, composite sum scores were calculated using only the retained items for each construct. These retained‐item composite scores were subsequently used in the mediation analyses.

After establishing measurement validity, composite (sum) scores of retained items were calculated and used in subsequent analyses. Pearson correlation coefficients were computed to examine bivariate associations among variables. Finally, mediation analysis was performed using the PROCESS macro (Model 4) with 5000 bootstrap resamples (Hayes and Scharkow 2013). Work Capital (WC) was specified as the independent variable, decent work (DW) as the mediator and work engagement (UWES) as the dependent variable. Demographic variables (age, work years, education and professional title) were included as covariates to enhance robustness. Although CFA was conducted within a latent‐variable framework, mediation analysis was performed using observed composite scores to obtain bias‐corrected bootstrap estimates of indirect effects, consistent with widely used approaches in applied research. To further ensure robustness, a supplementary latent‐variable mediation model using structural equation modelling (SEM) yielded consistent results (Table S1).

## 4 Results

### 4.1 Sample Characteristics

A total of 535 nurses were included in the final analysis (response rate = 90.2%). The sample was predominantly female (96.1%), reflecting the gender distribution of the nursing workforce. The largest age group was 31–39 years (53.3%), followed by 40–49 years (21.5%). In terms of professional characteristics, most participants held a bachelor's degree (92.7%), and over half were nurse practitioners‐in‐charge (54.0%), indicating a relatively experienced workforce. Work experience was broadly distributed, with 27.3% reporting 11–15 years of experience. Participants were recruited from multiple departments, including medical, surgical, intensive care and emergency units, providing variability in clinical contexts (Table 1).

**TABLE 1:** Demographic Characteristics of the Nurses (*N* = 535).

Age	Frequency	Percentage (%)
< 30	120	22.4
31 ~ < 40	285	53.3
41 ~ < 50	115	21.5
51 ~ < 60	14	2.6
> 60	1	0.2
Education		
Bachelor's degree	496	92.7
Master's degree or PhD	39	7.3
Title		
Nurse	28	5.2
Senior nurse	155	29
Nurse practitioner‐in‐charge	289	54
Associate or chief nurse practitioner	63	11.8
Work years		
< 6	72	13.5
6 ~ < 11	152	28.4
11 ~ < 16	146	27.3
16 ~ < 21	94	17.6
> 21	71	13.3
Sex		
Male	21	3.9
Female	514	96.1

### 4.2 CFA Model Fit and Measurement Instruments

A confirmatory factor analysis (CFA) was conducted to evaluate the three‐factor measurement model (WC, DW, UWES). The initial model demonstrated suboptimal fit (CFI = 0.88, TLI = 0.86, RMSEA = 0.072), indicating the need for model refinement. Items with standardized factor loadings below 0.60 or high residuals were examined. Removal decisions were guided by both statistical criteria and theoretical considerations to preserve construct meaning. This process resulted in a refined 35‐item model (UWES = 11, DW = 11, WC = 13). The revised model demonstrated acceptable fit: χ^2^(735) = 1643.27, *p* < 0.001; χ^2^/df = 2.24; CFI = 0.92; TLI = 0.91; RMSEA = 0.056 (90% CI [0.050, 0.062]); SRMR = 0.046. The AVE values for work capital, decent work and work engagement were 0.61, 0.67, and 0.70, respectively, all exceeding the recommended threshold of 0.50. Furthermore, the square roots of the AVEs were greater than the inter‐construct correlations, satisfying the Fornell–Larcker criterion for discriminant validity. HTMT ratios ranged from 0.72 to 0.84, below the recommended cutoff of 0.85, further supporting adequate construct distinctiveness.

### 4.3 Descriptive Statistics and Correlations

The descriptive statistics and correlations are presented in Table 2. The total scores were 85.54 (SD = 17.23) for work capital, 78.97 (SD = 17.06) for decent work, and 69.51 (SD = 24.39) for work engagement. Work capital was positively correlated with decent work (*r* = 0.76, *p* < 0.001) and work engagement (*r* = 0.62, *p* < 0.001). Decent work was also positively correlated with work engagement (*r* = 0.73, *p* < 0.001).

**TABLE 2:** Descriptive Statistics and Correlations Among Study Variables (*N* = 535).

Variable	Mean	SD	1	2	3	4	5	6	7	8	9	10	11	12	13	14	15
WC	85.54	17.23	1														
WC_EWC	21.34	5	0.888**	1													
WC_HWC	22.56	4.25	0.884**	0.72**	1												
WC_SWC	19.32	6	0.885**	0.73**	0.65**	1											
WC_CWC	22.32	4.287	0.865**	0.66**	0.79**	0.64**	1										
UWES	69.51	24.39	0.62**	0.51**	0.56**	0.54**	0.58**	1									
UWES_V	23.2	8.48	0.60**	0.50**	0.55**	0.52**	0.57**	0.97**	1								
UWES_A	14.24	5.22	0.55**	0.47**	0.52**	0.49**	0.54**	0.94**	0.90**	1							
UWES_D	32.07	11.31	0.62**	0.51**	0.55**	0.55**	0.58**	0.98**	0.94**	0.90**	1						
DW	78.97	17.06	0.76**	0.64**	0.74**	0.63**	0.71**	0.73**	0.72**	0.66**	0.73**	1					
DW_WES	16.66	3.42	0.73**	0.60**	0.70**	0.58**	0.71**	0.60**	0.59**	0.54**	0.61**	0.85**	1				
DW_HBP	16.97	3.49	0.67**	0.54**	0.71**	0.50**	0.66**	0.59**	0.58**	0.54**	0.58**	0.88**	0.78**	1			
DW_PFA	15.04	4.1	0.67**	0.59**	0.62**	0.58**	0.59**	0.67**	0.66**	0.62**	0.67**	0.91**	0.70**	0.79**	1		
DW_WLF	14.31	4.47	0.64**	0.53**	0.60**	0.57**	0.57**	0.64**	0.63**	0.57**	0.64**	0.86**	0.62**	0.63**	0.75**	1	
DW_VA	15.98	3.81	0.67**	0.56**	0.66**	0.52**	0.63**	0.71**	0.70**	0.66**	0.70**	0.89**	0.72**	0.74**	0.77**	0.72**	1

*Note:* All scale scores are calculated as the sum of retained items, with higher scores indicating higher levels of the respective constructs. Pearson correlation coefficients are reported. Significance levels: *p < 0.05, **p < 0.01, ***p < 0.001.

Abbreviations: DW, decent work total score; UWES, work engagement total score; UWES_A, work engagement absorption; UWES_D, work engagement dedication; UWES_V, work engagement vigour; WC, work capital total score; WC_CWC, cultural work capital; WC_EWC, economic work capital; WC_HWC, human work capital; WC_SWC, social work capital; WD_HBP, healthcare benefit provision; WD_PFA, pay fairness and adequacy; WD_VA, value alignment; WD_WES, workplace emotional safety and absence of abuse; WD_WLF, work‐life balance and leisure time.

### 4.4 Hypothesis Testing for Pathways

A supplementary sensitivity analysis using the original unreduced scale scores yielded comparable mediation results, with consistent directions and magnitudes of direct and indirect associations (Table S2). These findings suggest that the observed mediation pathway was robust to the item‐refinement procedure (Table 3). The results showed that work capital was positively associated with decent work (a path: B = 0.74, SE = 0.03, *p* < 0.001). In turn, decent work was positively associated with work engagement (b path: B = 0.85, SE = 0.06, *p* < 0.001). The total association between work capital and work engagement (c path) was significant (B = 0.82, SE = 0.05, 95% CI [0.72, 0.92], *p* < 0.001). After including decent work in the model, the direct association between work capital and work engagement (c'path) remained statistically significant but was reduced in magnitude (B = 0.19, SE = 0.05, 95% CI [0.08, 0.30], *p* < 0.001). Bootstrap analysis indicated that the indirect association of work capital with work engagement through decent work was statistically significant (indirect effect = 0.63, BootSE = 0.05, 95% CI [0.53, 0.74]). These findings suggest that decent work may function as a potential explanatory pathway linking work capital and work engagement. The proportion mediated was calculated as the ratio of the indirect effect to the total effect (0.63/0.82 = 76.8%). However, this value should be interpreted cautiously because proportion‐mediated estimates in cross‐sectional models do not establish causal mediation and may be sensitive to model specification. The overall regression models demonstrated acceptable explanatory power (DW model: adjusted *R* ^2^ = 0.57, F[5529] = 143.27, *p* < 0.001; UWES model: adjusted *R* ^2^ = 0.64, F[6528] = 157.84, *p* < 0.001). The proportion mediated was estimated descriptively as the ratio of the indirect association to the total association. However, because the present study employed a cross‐sectional design, this value should not be interpreted as evidence of causal mediation. Moreover, proportion‐mediated estimates may vary substantially across alternative model specifications, measurement strategies and included covariates.

**TABLE 3:** Mediation effect of DW between WC and UWES.

	DW				UWES			
	*B*	SE	*t*	*P*	*B*	SE	*t*	*p*
WC	0.76	0.02	27.7	< 0.001	0.21	0.06	3.26	< 0.001
DW					0.88	0.06	13.6	< 0.001
Adjust *R* ^2^	0.54				0.59			
F	322.08				767.39			
WC→DW→UWES	Effect		BootSE		95% CI			

					Low Bound		Upper Bound	
Indirect Effect	0.67		0.05		0.56		0.79	
Direct Effect	0.21		0.06		0.07		0.34	
Total Effect	0.88		0.04		0.71		0.81	

*Note:* Mediation analyses were conducted using composite sum scores derived from retained items after CFA refinement.

Abbreviations: WC, work capital; DW, decent work; UWES, Work Engagement.

## 5 Discussion

### 5.1 Nurses' Work Engagement at a Moderate Level: Lowest in Dedication, Highest in Absorption

The present study identified moderate‐to‐high levels of work engagement among nurses. Comparisons across vigour, dedication and absorption were conducted using standardized mean scores because the UWES dimensions contain different numbers of items. After standardization, dedication demonstrated relatively higher levels, whereas absorption appeared comparatively lower. Previous investigations in tertiary hospitals in China have similarly reported those results (Wan et al. 2018; Xu, Lin, et al. 2024; Zhang et al. 2025). The deficiency in dedication may be attributed to low social recognition of the nursing role, heightened risk of burnout and long‐term exposure to stressful work environments and night shifts (Jenaro et al. 2011). In contrast, the absorption dimension scored the highest, suggesting that nurses maintain a high level of focus while performing clinical tasks. This finding aligns with Schaufeli et al. (2002), who identified absorption as a critical psychological resource for coping with job stress. Compared with some studies conducted in Western healthcare settings, the nurses in the present study demonstrated relatively stable levels of professional commitment and involvement. This pattern may be related to differences in clinical workload, occupational expectations and healthcare environments (Wu et al. 2022).

### 5.2 Work Capital Positively Influences Decent Work Among Nurses

The results demonstrated that nurses' work capital significantly enhanced their perception of decent work. This finding supports the resource gain assumption of the JD‐R model, which posits that individuals with greater psychological and social capital are more likely to perceive their work as fair, secure and developmental. Consistent with the findings of Yu and Zeng.

Nurses with higher levels of Work Capital tended to report more positive perceptions of decent work. This finding suggests that workplace‐related resources, including economic, human, social and cultural resources, may be associated with how nurses evaluate the fairness, safety and quality of their work environments. Similar findings have been reported in previous occupational studies showing that supportive workplace resources are associated with more positive evaluations of work conditions (Zheng et al. 2025; Yu et al. 2024). Those prior studies often focused narrowly on psychological capital. In this study, work capital was discussed as a workplace‐resource construct, whereas decent work reflected nurses' perceptions of work quality and dignity. Therefore, nursing administrators should adopt integrated strategies, including skill development programs, fostering team‐based support systems and establishing positive feedback mechanisms to strengthen nurses' perceptions of decent work (Yu et al. 2024; Fang et al. 2025).

### 5.3 Work Capital Positively Influences Nurses' Work Engagement

This study further validated the motivational pathway proposed by the JD‐R model: work capital, as a critical job resource, directly promotes nurses' work engagement. This finding is consistent with Bakker et al. (2014), who argued that the accumulation of personal and organizational resources enhances motivational processes, thereby increasing engagement. Prior research also supports this conclusion. Xue and Zhang found that psychological capital effectively mitigates burnout and enhances nurses' engagement (Zhang et al. 2023; Xue et al. 2023). Unlike studies focusing mainly on psychological capital, the present study conceptualized work capital more broadly to include economic, human, social and cultural resources. In addition, the association between work capital and work engagement remained statistically significant after controlling for decent work and demographic variables, suggesting that work capital retained an independent relationship with nurses' engagement levels.

### 5.4 The Mediating Role of Decent Work Between Work Capital and Work Engagement

The present study indicated that decent work played an important role in the relationship between work capital and work engagement. Nurses with higher levels of work capital also tended to report more positive perceptions of decent work, which was further associated with higher levels of work engagement. Because work capital and decent work are both theoretically connected to workplace resources, potential conceptual overlap warrants consideration, particularly after CFA‐based item refinement. However, discriminant validity analyses supported the distinction between the constructs. Conceptually, work capital primarily reflects the availability of organizational and occupational resources, whereas decent work represents employees' subjective appraisal of fairness, dignity and quality of work conditions. Thus, although related, the constructs capture different levels of workplace experience within the JD‐R framework. This finding is consistent with the JD‐R framework, suggesting that workplace‐related resources may contribute to motivational processes through employees' perceptions of their work environments (Allan et al. 2019). Unlike previous studies, the present study further highlighted the occupational characteristics of nursing, in which Decent Work was closely associated with job stability, professional dignity and supportive work environments. Therefore, both individual resource development and organizational support may be important for promoting nurses' work engagement and professional development (Zheng et al. 2025). However, because the present study was cross‐sectional, the observed mediation pathway should be interpreted cautiously. Alternative explanations may also exist. Nurses with higher work engagement may perceive their work conditions more positively and report higher levels of work capital and decent work.

### 5.5 Theoretical And Practical Implications

This study used the JD‐R model by introducing decent work as a mediating mechanism between work capital and work engagement in the nursing context. The findings suggest that workplace‐related resources and perceptions of work conditions may both be associated with nurses' occupational engagement. From a practical perspective, the findings suggest that healthcare organizations may benefit from strengthening workplace‐resource support systems, including professional development opportunities, supportive team environments and fair organizational management practices. In addition, improving nurses' perceptions of work quality, dignity and organizational support may help maintain professional engagement and sustainable occupational development. Although the indirect association accounted for a substantial proportion of the total association, this estimate should be interpreted cautiously. In cross‐sectional mediation models, proportion‐mediated values are descriptive rather than causal and may be highly sensitive to model specification, omitted variables and measurement approaches. Therefore, the present findings should be regarded as preliminary evidence of a potential explanatory pathway rather than confirmation of a definitive mediational mechanism.

### 5.6 Limitations

This study has four primary limitations. First, its cross‐sectional design limits the ability to infer causality; future research may employ longitudinal or experimental designs to validate the dynamic relationships among variables. The sample was drawn from a single province, which may limit generalizability. Due to the high proportion of female and bachelor‐level participants, subgroup comparisons were limited. Second, participants were drawn mainly from specific regions or hospitals, which may restrict representativeness and limit the generalizability of the findings to nurses across different settings and levels. Third, data were collected through self‐reported questionnaires, which may be subject to social desirability bias or subjective responses. Future studies could incorporate multisource data, such as supervisor assessments or peer feedback, to enhance objectivity. Finally, although this study examined work capital, decent work, and work engagement, it did not include other potentially relevant factors such as job demands, emotional labor or organizational support. Subsequent research should expand the model to provide a more comprehensive understanding of nurses' work‐related psychological mechanisms.

## 6 Conclusion

The present study demonstrated significant associations among work capital, decent work and work engagement among nurses. Decent work was also statistically associated with the relationship between work capital and work engagement. These findings provide empirical support for the applicability of the JD‐R framework in understanding nurses' occupational engagement. From a practical perspective, the findings suggest that workplace‐related resources and supportive work environments may be relevant to maintaining nurses' professional engagement and occupational development.

## Author Contributions

L.L. and Q.Z. contributed to the conceptualization, and writing – original draft. Q.Z., Y.L., T.Z., L.L.Y., J.Y., L.H.Y., L.L., S.Z. and Q.Z. contributed to the conceptualization, data curation, formal analysis, funding acquisition, investigation, methodology, project administration, software, resources, supervision, validation, visualization, writing – original draft, writing – review and editing.

## Funding

The authors have nothing to report.

## Ethics Statement

Hangzhou Linping District Integrated Traditional Chinese and Western Medicine Hospital (Approval no. 2025‐L‐029) approved this study. We explained the purpose of the study to the participants before distributing the questionnaire. Additionally, we ensured that participants were voluntary and anonymous and allowed them to opt out at any time. We stored the data securely, and only research team members had access to, stored, and processed the data. All participants signed the informed consent form and completed the questionnaires.

## Consent

The authors have nothing to report.

## Conflicts of Interest

The authors declare no conflicts of interest.

## Supporting information


**Table S1:** Latent‐variable mediation model using structural equation modelling.

**Table S2:** Sensitivity analysis using the original unreduced scale scores yielded comparable mediation results, with consistent directions and magnitudes of direct and indirect associations.

## Data Availability

The data that support the findings of this study are available from the corresponding author upon reasonable request.
