# Supplementary material for: Decent Work Mediates the Relationship Between Work Capital and Work Engagement Among Nurses
Source: Nurs Open. 2026 Jun 8;13(6):e70633. doi: 10.1002/nop2.70633 (PMC13246464; doi:10.1002/nop2.70633)
Supplement: Supplementary file 1 — Table S1: Latent‐variable mediation model using structural equation modelling. [file NOP2-13-e70633-s001.docx]

Supplementary Table S1. Item Refinement Results for UWES, DW, and WC

| **Scale** | **Item** | **Retained (Yes/No)** | **Standardized Loading (CFA)** | **Reason for Removal/Retention** | **Cronbach’s α (Retained Items)** |
| --- | --- | --- | --- | --- | --- |
| UWES | UWES1 | Yes | 0.84 | Retained | 0.92 |
|  | UWES2 | Yes | 0.86 | Retained |  |
|  | UWES3 | No | 0.60 | Marginal loading and limited model contribution |  |
|  | UWES4 | Yes | 0.90 | Retained |  |
|  | UWES5 | Yes | 0.89 | Retained |  |
|  | UWES6 | No | 0.59 | Low loading |  |
|  | UWES7 | Yes | 0.82 | Retained |  |
|  | UWES8 | Yes | 0.86 | Retained |  |
|  | UWES9 | No | 0.58 | Low loading |  |
|  | UWES10 | Yes | 0.86 | Retained |  |
|  | UWES11 | Yes | 0.76 | Retained |  |
|  | UWES12 | No | 0.57 | Low loading |  |
|  | UWES13 | Yes | 0.87 | Retained |  |
|  | UWES14 | Yes | 0.77 | Retained |  |
|  | UWES15 | Yes | 0.85 | Retained |  |
| DW | DW1 | Yes | 0.82 | Retained | 0.93 |
|  | DW2 | No | 0.55 | Low loading |  |
|  | DW3 | Yes | 0.76 | Retained |  |
|  | DW4 | Yes | 0.85 | Retained |  |
|  | DW5 | No | 0.57 | Low loading |  |
|  | DW6 | Yes | 0.86 | Retained |  |
|  | DW7 | Yes | 0.87 | Retained |  |
|  | DW8 | Yes | 0.83 | Retained |  |
|  | DW9 | Yes | 0.85 | Retained |  |
|  | DW10 | No | 0.57 | Low loading |  |
|  | DW11 | Yes | 0.76 | Retained |  |
|  | DW12 | Yes | 0.74 | Retained |  |
|  | DW13 | No | 0.55 | Low loading |  |
|  | DW14 | Yes | 0.88 | Retained |  |
|  | DW15 | Yes | 0.84 | Retained |  |
| WC | WC1 | Yes | 0.68 | Retained | 0.91 |
|  | WC2 | Yes | 0.77 | Retained |  |
|  | WC3 | Yes | 0.74 | Retained |  |
|  | WC4 | No | 0.58 | Low loading |  |
|  | WC5 | Yes | 0.85 | Retained |  |
|  | WC6 | Yes | 0.82 | Retained |  |
|  | WC7 | No | 0.58 | Localized strain identified through modification indices |  |
|  | WC8 | Yes | 0.86 | Retained |  |
|  | WC9 | Yes | 0.76 | Retained |  |
|  | WC10 | Yes | 0.76 | Retained |  |
|  | WC11 | Yes | 0.81 | Retained |  |
|  | WC12 | Yes | 0.75 | Retained |  |
|  | WC13 | Yes | 0.84 | Retained |  |
|  | WC14 | Yes | 0.83 | Retained |  |
|  | WC15 | No | 0.57 | Low loading |  |
|  | WC16 | Yes | 0.80 | Retained |  |

Note: Item refinement was conducted based on overall model fit, factor loadings, modification indices, and conceptual relevance. Items with low or marginal loadings and limited contribution to model stability were considered for removal during CFA refinement. Cronbach’s coefficients for retained items were 0.91 (WC), 0.93 (DW), and 0.92 (UWES), indicating acceptable internal consistency after refinement. Standardized mean scores were used for subdimension comparisons where applicable because retained item numbers differed across dimensions
