# Supplementary material for: Decent Work Mediates the Relationship Between Work Capital and Work Engagement Among Nurses
Source: Nurs Open. 2026 Jun 8;13(6):e70633. doi: 10.1002/nop2.70633 (PMC13246464; doi:10.1002/nop2.70633)
Supplement: Supplementary file 2 — Table S2: Sensitivity analysis using the original unreduced scale scores yielded comparable mediation results, with consistent directions and magnitudes of direct and indirect associations. [file NOP2-13-e70633-s002.docx]

Supplementary Table S2. Sensitivity Analysis Using Original Full-Scale Scores Before Item Deletion

| **Pathway** | **B** | **SE** | **95% CI** | **p** |
| --- | --- | --- | --- | --- |
| WC → DW (a path) | 0.75 | 0.03 | [0.69, 0.81] | < .001 |
| DW → UWES (b path) | 0.86 | 0.06 | [0.74, 0.98] | < .001 |
| WC → UWES (total effect, c path) | 0.84 | 0.05 | [0.74, 0.94] | < .001 |
| WC → UWES (direct effect, c’ path) | 0.20 | 0.05 | [0.09, 0.31] | < .001 |
| Indirect effect (WC → DW → UWES) | 0.64 | 0.05 | [0.54, 0.75] | < .001 |

Note: Sensitivity analyses were conducted using the original unreduced full-scale composite scores prior to CFA-based item refinement. Results were highly consistent with the primary mediation analyses based on retained items, indicating that the observed indirect association was robust to the item-refinement procedure. Bootstrap confidence intervals were generated using 5,000 resamples. WC = Work Capital; DW = Decent Work; UWES = Work Engagement.
